# ID30A-3 (MASSIF-3) – a beamline for macromolecular crystallography at the ESRF with a small intense beam

**DOI:** 10.1107/S1600577520004002

**Published:** 2020-04-29

**Authors:** David von Stetten, Philippe Carpentier, David Flot, Antonia Beteva, Hugo Caserotto, Fabien Dobias, Matias Guijarro, Thierry Giraud, Mario Lentini, Sean McSweeney, Antoine Royant, Sebastien Petitdemange, Jeremy Sinoir, John Surr, Olof Svensson, Pascal Theveneau, Gordon A. Leonard, Christoph Mueller-Dieckmann

**Affiliations:** a European Synchrotron Radiation Facility, 71 Avenue des Martyrs, 38000 Grenoble, France; b Institut de Biosciences et Biotechnologies de Grenoble (BIG) – Laboratoire Chimie et Biologie des Métaux (LCBM), Université Grenoble Alpes, CNRS, CEA, 38000 Grenoble, France; c Institut de Biologie Structurale (IBS), Université Grenoble Alpes, CEA, CNRS, Grenoble, France; d EMBL Outstation Grenoble, 71 Avenue des Martyrs, 38000 Grenoble, France

**Keywords:** ID30A-3, MASSIF-3, high-brilliance, mini-focus, automation, macromolecular crystallography

## Abstract

ID30A-3 (or MASSIF-3) is a mini-focus (beam size 18 µm × 14 µm) highly intense (2.0 × 10^13^ photons s^−1^), fixed-energy (12.81 keV) beamline for macromolecular crystallography (MX) experiments at the European Synchrotron Radiation Facility (ESRF). MASSIF-3 is one of two fixed-energy beamlines sited on the first branch of the canted undulator setup on the ESRF ID30 port and is equipped with a MD2 micro-diffractometer, a Flex HCD sample changer, and an Eiger X 4M fast hybrid photon-counting detector. MASSIF-3 is recommended for collecting diffraction data from single small crystals (≤15 µm in one dimension) or for experiments using serial methods. The end-station has been in full user operation since December 2014, and here its current characteristics and capabilities are described.

## Abstract 2

ID30A-3, a highly intense mini-beam facility for macromolecular crystallography at the ESRF, is described..

## 1. Introduction   

In 2008, the ESRF launched Phase I of its upgrade program, an integral part of which was the replacement and modernization, as part of the ‘Upgrade Program Beamline 10’ (UPBL10; Theveneau *et al.*, 2013 ▸) project, of the ageing ID14 complex with a new suite of end-stations for structural biology. The experience gained from the operation of ID14 (*e.g.* diamond beam splitters; Wakatsuki *et al.*, 1998 ▸) and of ID23 (canted straight section with two independent X-ray sources; Nurizzo *et al.*, 2006 ▸; Flot *et al.*, 2010 ▸) helped to design state-of-the-art end-stations. The UPBL10 project produced three beamlines for macromolecular crystallography (MX) in a canted configuration [ID30A-1 (MASSIF-1; Bowler *et al.*, 2015 ▸) and ID30A-3 (MASSIF-3) on the ID30A branch, and ID30B (McCarthy *et al.*, 2018 ▸) on the other branch] as well as a beamline, BM29 (Pernot *et al.*, 2013 ▸), dedicated to BioSAXS. Locating UPBL10 close to ID29 (de Sanctis *et al.*, 2012 ▸) created an ‘MX Village’, facilitating the common usage of ancillary technique laboratories and sharing services including the *ic*OS *in crystallo* optical spectroscopy facility (von Stetten *et al.* 2015 ▸), a laboratory for the high-pressure cryo-cooling of biological macromolecules (HPMX; Lafumat *et al.*, 2016 ▸; van der Linden *et al.*, 2014 ▸), sample storage facilities both at 20°C and at cryogenic temperature, a sample preparation laboratory, and a data processing and backup computing area.

Conceived when CCD-based detectors were the staple device employed on synchrotron-based MX facilities, the original idea for the ID30A (MASSIF) beamlines was to create a hub of highly automated fixed-energy beamlines for the rapid characterization and sorting of samples. The best samples would then be redirected to the most appropriate ESRF beamlines [*i.e.* ID23-1 (Nurizzo *et al.*, 2006 ▸), ID23-2 (Flot *et al.*, 2010 ▸), ID29 (de Sanctis *et al.*, 2012 ▸), ID30B (McCarthy *et al.*, 2018 ▸)] for the much more time-consuming step of the optimized collection of full diffraction data sets. However, the advent of fast hybrid photon-counting detectors and shutterless data collection (Förster *et al.*, 2019 ▸) greatly reduced the time needed for the collection of high-quality diffraction data sets and therefore this concept was modified. MASSIF-1 is now a beamline dedicated to completely automatic MX experiments (Bowler *et al.*, 2015 ▸). MASSIF-3, described here, evolved into a mini-beam end-station equipped with an MD2 micro-diffractometer (Perrakis *et al.*, 1999 ▸; Cipriani *et al.*, 2007 ▸), a Flex-HCD sample changer (McCarthy *et al.*, 2018 ▸) and an Eiger X 4M hybrid photon-counting detector (Casanas *et al.*, 2016 ▸) which can operate with frame rates of up to 750 Hz. In addition to being an excellent vehicle for standard MX experiments based around ESRF-developed automation, this setup has already proven very useful in the application of new diffraction data collection methods [*i.e.* MeshAndCollect (Zander *et al.*, 2015 ▸)] and room-temperature synchrotron serial crystallography (SSX) data collection using sample delivery systems such as the high-viscosity extruder (HVE) system developed by Botha *et al.* (2015 ▸).

## 2. Beamline overview   

### 2.1. X-ray source and optics hutches   

**Figure 1:**
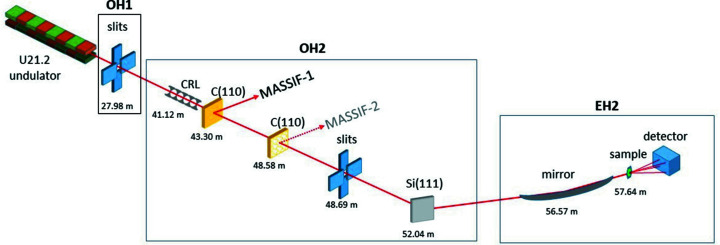
Schematic layout of the MASSIF-3 beamline. Major components are shown with their distance relative to the source. The monochromator/beamsplitter for MASSIF-2 is shown only for consistency but is currently not in place.

Before the extremely brilliant source upgrade (EBS; http://www.esrf.eu/about/upgrade) of the ESRF storage ring (see below), the X-ray source for the ID30A branch comprised two 1.4 m undulators with a periodicity of 21.2 mm (U21.2) operated in air with a minimum gap of 11 mm and optimized to emit their highest brilliance at an energy of 12.9 keV. A schematic overview of MASSIF-3 is shown in Fig. 1 ▸. There are two independent optical hutches (OH), OH1 that is shared with ID30B, and OH2 that contains most of the optical elements for the MASSIF beamlines. The canting angle between the sources for ID30A and ID30B is ±2.2 mrad, providing sufficient space between the two beams to install all optical equipment. Table 1 ▸ summarizes the current (and future, see below) beamline characteristics.

#### 2.1.1. OH1 configuration   

OH1 contains a set of ESRF standard high-power primary slits (Marion & Zhang, 2003 ▸), an ESRF high-vacuum white-beam viewer (wbv-a) and a photon absorber. The white-beam viewer is equipped with a Basler GigE camera that displays the fluorescence of a water-cooled CVD (chemical vapour deposition) diamond that can be inserted into the X-ray beam using a pneumatic actuator when this is required. The CVD diamond also acts as the scattering element for an integrated diode such that the intensity of the beam can be measured, for example, during alignment of the primary slits.

#### 2.1.2. OH2 configuration   

The MASSIF-specific OH2 houses most optical elements for the MASSIF beamlines as well as a long, lead-shielded vacuum tube which allows the white beam of the ID30B branch pass through the hutch. This setup ensures that ID30B is not affected in case of an intervention in OH2.

The first element in OH2 is a set of water-cooled cylindrical white-beam compound refractive lenses (CRLs, Snigirev *et al.*, 1996 ▸) made from beryllium. This device focuses the white beam vertically, producing a vertical beam size of 15 µm at the sample position (EH2, see below) of MASSIF-3. A synthetic asymmetric Laue [110]-cut diamond, 250 µm thick and mounted on a water-cooled copper support, is then used as a monochromator for MASSIF-1, deviating X-rays with an energy of 12.834 keV into its experimental hutch (EH1; Bowler *et al.*, 2015 ▸). The advantage of the Laue [110] geometry is that the Bragg planes diffracting X-rays are almost 90° relative to the diamond surface, which maximizes the transmission of the white beam (deprived of its 12.834 keV component). The transmitted white beam then impinges on a liquid-nitro­gen-cooled single Si(111) crystal with a bandwidth of 1.5 × 10^−4^ used as a monochromator for MASSIF-3, which delivers a beam at a photon energy of 12.812 keV into its experimental hutch (EH2). This energy on the high-energy remote side of the selenium edge was chosen in order to be able to use the anomalous scattering contribution from selenium for *de novo* phasing experiments. Immediately downstream of the monochromator, an energy calibration device has been installed. This contains a wheel with windows for up to six different pure metal foils and, coupled with a retractable direct diode, read by a Keithley picoammeter, allows for easy, automated energy calibration by recording of X-ray absorption spectra. Currently, the scanning of the *L*_II_ absorption edge of an iridium foil (12.824 keV, 100 µm foil) is used for the (re)calibration of the MASSIF-3 monochromator to its nominal energy (12.812 keV). A second white-beam viewer (wbv-f), identical to that located in OH1, is installed before the Si(111) monochromator to facilitate the alignment of the beamline and for X-ray beam diagnostic purposes. A monochromatic beam viewer (mbv-31), consisting of a cerium-doped YAG screen that can be pneumatically inserted into the X-ray beam, has been installed after the monochromator. Here also, a Basler GigE camera allows to visualize the beam footprint on the YAG, which additionally serves as a scattering element for a diode (read by a Keithley picoammeter) to measure relative beam intensities.

### 2.2. EH2 configuration   

**Figure 2:**
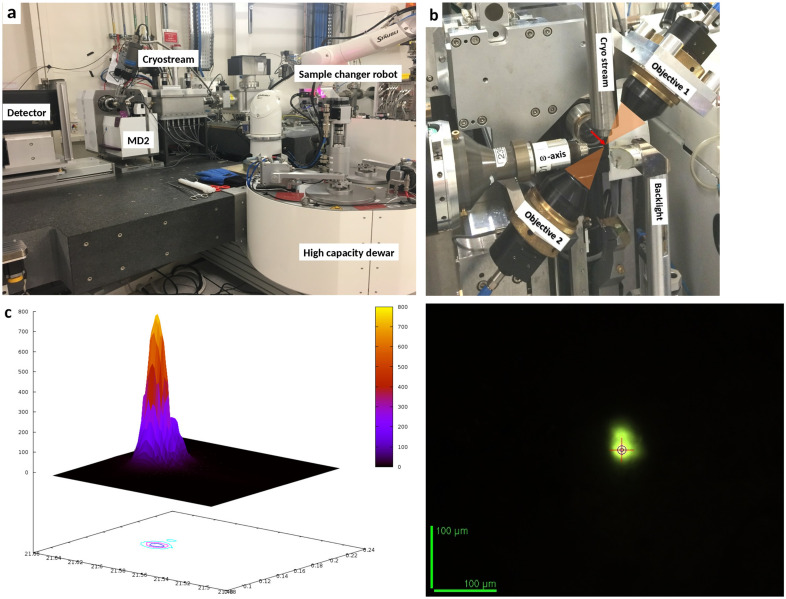
Experimental setup of MASSIF-3. (*a*) Photograph of the sample environment and (*b*) setup of the micro-spectrophotometer with the X-ray beam indicated as a red arrow and the optical pathway in transparent orange. (*c*) 3D plot of the X-ray beam profile measured with a 5 µm pinhole (abscissae in micrometres and ordinate in arbitrary units). (*d*) Image of the X-ray beam at the sample position as viewed through the on-axis viewer using the built-in scintillator (at 0.06% transmission and a storage ring current of 180 mA).

The experimental hutch of MASSIF-3 contains both the beamline’s horizontal focusing element and its sample position and sample environment. Horizontal focusing of the X-ray beam is achieved using a bent multilayer mirror (B_4_C/[Ru/B_4_C]_70_/Cr, manufactured in-house at ESRF) located 1.0 m upstream of the sample position. Combined with the vertical focusing provided by the white-beam CRLs (see above), this results in a focal spot size of 18 µm × 14 µm [FWHM as measured by scanning a tungsten wire across the beam; Fig. 2(*c*)] ▸ at the sample position. Potential contamination of diffraction intensities by X-ray photons of higher harmonic reflections from the Si(111) monochromator (mostly the third-harmonic reflections with photon energy ∼38.4 keV) are intrinsically suppressed or rejected due to the combination of three factors: (i) the X-ray detector’s quantum efficiency drastically decreases above 20 keV, (ii) the focusing distance of the CRLs depends on the photon energy such that higher energy photons are focused far behind the sample position, and (iii) the reflectivity of the multi-layer mirror drastically decreases at higher energies (Bigault *et al.*, 2003 ▸).

Upstream of the horizontally focusing mirror, a high-vacuum attenuator box containing a total of eight absorbing foils [two pyrocarbon (1 and 2 mm in thickness) and six Al (0.1, 0.2, 0.35, 0.5, 1, and 1.5 mm thickness)] is installed. This allows to accurately adjust the photon flux at the sample in small steps. Downstream of the mirror, a pair of beam-defining secondary slits (ss2) (JJ-XRAY, Lyngby, Denmark) has been installed to allow for the optimal alignment of the mirror and also to remove parasitic X-rays scattered from the mirror. This pair of slits is mounted in a ‘slitbox’ which also contains a scattering foil diode (*I*_0_), read by a Keithley picoammeter which allows constant monitoring of the X-ray beam intensity, a rotating millisecond fast shutter controlled by ESRF-developed IcePAP electronics (Janvier *et al.*, 2013 ▸), and a second scattering foil diode (*I*_1_) also read through a Keithley picoammeter which allows monitoring of the X-ray beam intensity after the fast shutter. As at the other ESRF Structural Biology Group MX beamlines, the *I*_0_ reading is correlated with photon flux using a calibrated diode periodically placed at the sample position, thus allowing the constant measurement of photon flux impinging of the sample.

### 2.3. Sample environment   

The MASSIF-3 sample environment [Fig. 2(*a*) ▸] comprises a MD2 micro-diffractometer (Arinax, Voreppe, France; permanent loan from EMBL Grenoble Outstation), an Eiger X 4M detector (Dectris, Baden-Daettwil, Switzerland; Casanas *et al.*, 2016 ▸; Förster *et al.*, 2019 ▸) mounted on a fast translation table, and a FlexHCD robotic sample changer (McCarthy *et al.*, 2018 ▸). Samples on the rotation axis of the MD2 are kept at cryogenic temperature using a 700 series cryo-stream (Oxford Cryostreams, Oxford, UK) equipped with an annealing system (Giraud *et al.*, 2009 ▸). The entire experimental setup [Fig. 2(*a*) ▸], including the slitbox described above, is installed on a large granite table to allow for the alignment of all elements relative to the X-ray beam using three vertical (tz1, tz2 and tz3) and two horizontal (tyf and tyb) motors. These motors are operated in a closed-loop feedback mode and can be moved independently, or conjointly as pseudo motors for controlling the table height (thgt), translation (ttrans), roll (txrot), pitch (tyrot), and yaw (tzrot). The MD2 micro-diffractometer is, in contrast to all other MX beamlines, not permanently equipped with a Mini-Kappa goniometer head (MK3, Brockhauser *et al.*, 2013 ▸) to keep the sphere of confusion to less than 1 µm. However, the mounting of an MK3 device is available upon request.

The maximum speed of the MD2 rotation axis is currently 720° s^−1^, which allows, in combination with the fast Eiger X 4M detector, the recording of complete datasets in less than a second (Table 2 ▸). Additional built-in accessories of the MD2 include beam-cleaning apertures and a YAG screen for automatic alignment of the rotation axis and the X-ray beam [Fig. 2(*d*) ▸]. The MD2 is equipped with a standard G-shaped beamstop/capillary setup, but optionally a motorized beamstop with a maximum distance of 120 mm from the sample can be mounted, leaving more space for experiments that require installation of specific setups that are significantly bulkier than standard SPINE pins. The FlexHCD robotic sample changer allows to use SPINE sample holders stored either in SC3 pucks (Cipriani *et al.*, 2006 ▸) or in UniPuck (http://smb.slac.stanford.edu/robosync/Universal_Puck) containers. Each system needs its own specific FlexHCD gripper to transfer sample holders, and the exchange of grippers can be performed automatically within less than a minute *via* the beamline control GUI *MXCuBE* (http://MXCuBE.github.io/MXCuBE; Gabadinho *et al.*, 2010 ▸; Oscarsson *et al.*, 2019 ▸).

The Eiger X 4M detector provides diffraction images in HDF5 format (https://portal.hdfgroup.org/display/support), which allows the storage of many (typically 100) images in a single file together with extensive metadata, thus reducing file system clutter. Most crystallographic software programs [*e.g.* *XDS* (Kabsch, 2010 ▸), *DIALS* (Winter *et al.*, 2018 ▸); *CrystFEL* (White *et al.*, 2012 ▸)] are able to handle this data format, but for certain steps of automatic data processing [namely strategy calculations using EDNA/BEST (Bourenkov & Popov, 2006 ▸; Incardona *et al.*, 2009 ▸)] diffraction images are automatically converted to miniCBF format (Bernstein & Hammersley, 2006 ▸). The maximum resolution that can be achieved with the Eiger X 4M at the shortest sample-detector distance available on MASSIF-3 is 1.52 Å at the edge of the detector, 1.19 Å at its corners.

MASSIF-3 is currently recommended for diffraction data collection from small crystals (<15 µm) or from larger crystals where use of a relatively small beam will allow the use of helical data collections (Flot *et al.*, 2010 ▸) or the identification of the best diffracting segment of the crystal for a diffraction experiment (*i.e.* diffraction cartography; Bowler *et al.*, 2010 ▸]). As on all other ESRF MX beamlines, a HC1 humidity device (Arinax, Moirans, France) that allows the recording of diffraction data under controlled relative humidity at room temperature is available upon user request (Sanchez-Weatherby *et al.*, 2009 ▸; Russi *et al.*, 2011 ▸). The beamline is also well adapted for experiments requiring the on-line collection of *in crystallo* fluorescence and/or UV/vis absorption spectra exploiting the use of a microspectrophotometer [McGeehan *et al.*, 2009 ▸; von Stetten *et al.*, 2015 ▸; Fig. 2 ▸(*b*)]. Additionally, MASSIF-3 is an excellent vehicle for serial synchrotron crystallography (SSX) experiments using a high-viscosity extruder (HVE; Botha *et al.*, 2015 ▸, results to be published elsewhere) in close collaboration with the group of Ilme Schlichting from the MPI for Medical Research in Heidelberg, Germany.

### 2.4. Beamline control and data collection   

Low-level control of all beamline components and communication with the PMAC controller of the MD2 is provided by the newly developed ESRF standard software *BLISS* (*Beamline Instrumentation Support Software*; https://gitlab.esrf.fr/bliss/bliss; Guijarro *et al.*, 2017 ▸). *BLISS* commands are mostly accessed transparently from the beamline control GUI *MXCuBE2* (Gabadinho *et al.*, 2010 ▸; Oscarsson *et al.*, 2019 ▸), but, for troubleshooting, a terminal interface is also available. All motors except those of the MD2 and that of the millisecond fast shutter are controlled by ESRF-developed IcePAP electronics (Janvier *et al.*, 2013 ▸) and pneumatically driven devices are controlled by WAGO control modules (https://www.wago.com).

**Figure 3:**
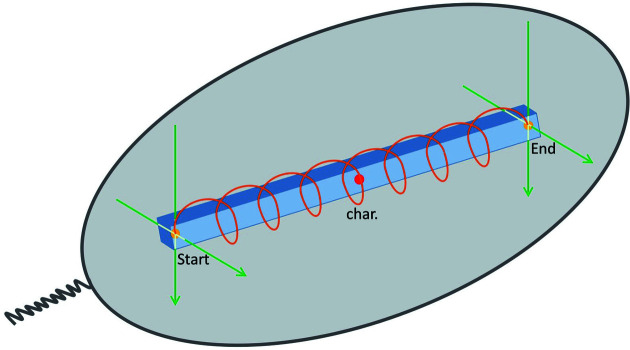
Schematic representation of the procedure for optimized helical data collection. Two orthogonal line scans (green arrows) at each end of the needle-shaped crystal (blue) precisely locate the start and end positions. Then a characterization is performed in the middle of the crystal (red dot), and the data set is collected along the crystal (orange line).

Users collect diffraction data *via* the *MXCuBE2* GUI (Gabadinho *et al.*, 2010 ▸; Oscarsson *et al.*, 2019 ▸) which is the standard interface on all MX beamlines at the ESRF. The metadata of each data collection are stored in the ISPyB database (Delagenière *et al.*, 2011 ▸) and can be recalled through the web-service EXI (https://exi.esrf.fr). Specific automated workflows are available and executed from within *MXCuBE* for complex tasks such as fast mesh scans, X-ray centring, helical data collections (Flot *et al.*, 2010 ▸), MeshAndCollect (Zander *et al.*, 2015 ▸), as well as MXpress workflows for fully automatic data collection (Svensson *et al.*, 2015 ▸; McCarthy *et al.*, 2018 ▸). MASSIF-3 also features a more advanced workflow for helical data collection (Fig. 3 ▸). This workflow is especially useful for cases where crystals are thin elongated needles or plates and where start and end points for the helical trajectory are difficult to assign due to the opaqueness and/or refraction (*i.e.* lensing effects) of frozen mother liquor. In this workflow, initial start/stop points for the trajectory are refined and precisely determined by X-ray diffraction using two orthogonal line scans at each point. A data collection strategy taking into account that the radiation damage will be distributed over a larger crystal volume (Zeldin *et al.*, 2013 ▸) is then calculated using characterization images collected at the midpoint of the trajectory and is subsequently executed (Fig. 3 ▸).

As on the other MX beamlines at the ESRF, all collected datasets are automatically processed using a series of software pipelines [*EDNAproc*, *GrenADeS* (Monaco *et al.*, 2013 ▸), *XDSAPP* (Sparta *et al.*, 2016 ▸), *DIALS* (Winter *et al.*, 2018 ▸), *autoPROC* (Vonrhein *et al.*, 2011 ▸)], the results of which are stored in the ISPyB database and are accessible, along with files containing the reduced data, through the web-service EXI (https://exi.esrf.fr; Delagenière *et al.*, 2011 ▸).

Data backup is easily handled, using an in-house-developed GUI that allows the users to select a specific beamline, experiment number, date, and USB drive, and to automatically copy their data in parallel to the experiment.

## 3. Results   

**Figure 4:**
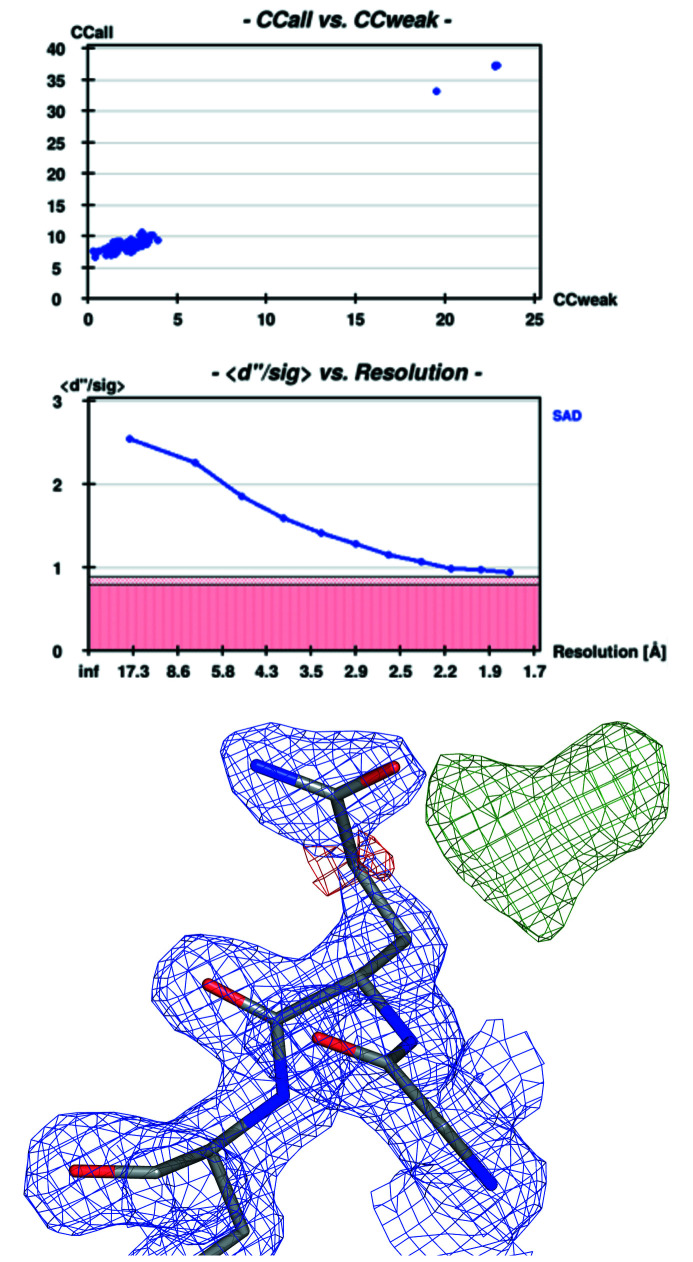
Indicators for substructure solution as output from *SHELXD* and a section of the resulting electron density map for the FAE diffraction data set. The top panel shows CC_all_ *versus* CC_weak_, the middle panel *d*′′/sig *versus* resolution, and the bottom panel the electron density [prepared with *PyMOL* v1.8 (Schrödinger, LLC) of residue Gln127 which exists in a double conformation (2*F*_o_ − *F*_c_) map at 1.5σ is shown in blue chicken wire, positive electron density for the minor conformation of residue 127 at 2.5σ in green, and the model in stick representation].

Opened to external users in December 2014, diffraction data collected on MASSIF-3 have, at the time of writing, contributed to 150 publications (source: ESRF-ILL Library; http://www.epn-campus.eu/1/library/the-joint-ill-esrf-library/) and have resulted in more than 150 depositions in the Protein Data Bank (PDB; wwPDB consortium, 2019 ▸; http://biosync.sbkb.org/stats.do?stats_sec=RGNL&stats_focus_lvl=SITE&stats_site=ESRF). Table 2 ▸ and Fig. 4 ▸ illustrate the high quality of data that can be routinely collected at the beamline.

In a first experiment, we collected diffraction data from a Se-Met derivatized crystal of ferulic acid esterase (FAE; Prates *et al.*, 2001 ▸) with the aim of *de novo* structure determination using the SAD technique (*f*′′ = 3.7 e^−^ at 12.834 keV). Data were measured according to a strategy proposed by an EDNA characterization (Incardona *et al.*, 2009 ▸) at 100 K from a crystal cryo-protected using Paratone N and were automatically processed (Monaco *et al.*, 2013 ▸). Subsequent *de novo* phasing performed with *hkl2map* (Pape & Schneider, 2004 ▸) and *SHELXC/D/E* (Usón & Sheldrick, 2018 ▸) found all 16 Se sites (8 per chain), and automatic building with *ARP/WARP* (Langer *et al.*, 2008 ▸) resulted in a model containing 280 and 281 residues (out of 297 residues) for the two FAE monomers in the asymmetric unit which was then further refined using *Coot* (Emsley *et al.*, 2010 ▸) and *refmac5* (Kovalevskiy *et al.*, 2018 ▸). A representative section of electron density is shown in Fig. 4 ▸ which illustrates OMIT difference density for a double conformation for residue Gln127 (corresponding to Gln915 in PDB access code 1gkk).

In a second experiment, bovine trypsin purchased as lyophilized powder from Sigma (reference: T9201) was crystallized as follows: A solution of 40 mg ml^−1^ trypsin, 10 mg ml^−1^ benzamidine (Sigma B-6506) and 3 m*M* CaCl_2_ was mixed in a 1:1 ratio with reservoir solution [2 *M* (NH_4_)_2_SO_4_ in 100 m*M* Tris-HCl pH 8.5] at room temperature. Crystals appeared after a few days and were cryo-protected using 20% (*w*/*v*) glycerol. Diffraction data were then collected at 100 K using two different strategies: the first based on a strategy provided by EDNA/BEST (500 imgages s^−1^, 50° s^−1^ omega rotation, total data collection time 2.8 s, trypsin-EDNA in Table 2 ▸), the second using a manually determined strategy optimized for data collection speed (746 images s^−1^, 149° s^−1^ omega rotation, total data collection time 0.93 s, trypsin-fast in Table 2 ▸). While the ‘slow’ diffraction data set has been collected to show a standard data collection, the ‘fast’ diffraction data set has been collected to show the potential of the beamline. As can be seen in Table 2 ▸, the two data sets are of comparable quality (automatic processing using *EDNAproc*) with both giving clear structure solutions, requiring only little additional refinement (adding waters, benzamidine and other buffer molecules, adjusting side chains, *etc.*), using the ESRF automatic molecular replacement pipeline (Monaco *et al.*, 2013 ▸). The structure refinement based on the trypsin-fast dataset (PDB access code 6swv) demonstrates that on MASSIF-3 complete, high-quality diffraction datasets can be collected in less than a second (Table 2 ▸). The combination of the fast read-out detector and a fast rotation goniometer (up to 720° s^−1^) allows collection of diffraction data at room temperature from crystals (Gotthard *et al.*, 2019 ▸). The combination of a few dozen data wedges derived from different diffraction data sets allows the collection of time-resolved data in the millisecond range (Aumonier *et al.*, submitted).

## 4. Post ESRF-EBS configuration   

The ESRF-EBS upgrade, which will be completed in the autumn of 2020, has a number of implications for MASSIF-3. Notable amongst these (Table 1 ▸) will be a reduction in the horizontal source size (FWHM) from 412 µm to 28 µm while the vertical source size will stay the same. The horizontal source divergence will also be reduced from 11.5 µrad to 7.2 µrad. As described above, horizontal focusing on MASSIF-3 is currently achieved using a bent multilayer mirror, a choice that was made in order to accept the full horizontal footprint (approximately 76 mm) of the X-ray beam 56 m from the source. Post-ESRF-EBS the reduced horizontal footprint will make it possible to replace the multilayer mirror by a set of horizontally focusing CRLs, further simplifying the beamline setup and operation.

The upgrade of the ESRF storage ring also requires a reduction in the length of the ID30 straight section. As a result, the current ID30A X-ray source (2 × 1.4 m U21.2 undulators) will be replaced by a single U21.2 device 2.3 m in length. This, coupled with the acceptance of the entire X-ray beam by the vertically focusing white-beam CRLs aperture situated in OH1, should result in a more than twofold increase in the available flux at the sample position (*i.e.* ∼4 × 10^13^ photons s^−1^). Thus, while our current aim is to maintain a MASSIF-3 focal spot size of ∼15 µm in diameter, post-EBS the end-station should become an even more attractive vehicle for MX.

## 5. Conclusion   

ID30A-3 (MASSIF-3) is a fixed-energy (12.81 keV), highly intense (>10^13^ photons s^−1^) and highly automated mini-beam (18 µm × 14 µm) end-station dedicated to protein crystallography at the ESRF. The beamline has been in full user operation since December 2014 with, at the time of writing, data collected at the beamline contributing to more than 150 PDB depositions (https://www.rcsb.org; Berman *et al.*, 2003 ▸) and more than 150 publications (https://epn-library.esrf.fr). MASSIF-3 is currently recommended for diffraction data collection from small crystals or from larger crystals where use of a relatively small beam will allow the use of helical data collections or the identification of the best diffracting segment of a crystal for a diffraction experiment. Moreover, the relatively small focal spot size is conducive to efficient, high-quality experiments exploiting multi-sample or serial methods. Post-ESRF-EBS, simplification of the beamline’s optical setup coupled with an increase in the available flux at the sample position will ensure that the end station remains an extremely attractive vehicle for MX in the coming decade.

## Figures and Tables

**Table 1:** Beamline parameters of ID30A-3 (MASSIF-3)

	2014–2019	From 2020
Source	2 × 1.4 m U21.2 in-air undulators (gap setting: 11 mm)	1 × 2.3 m U21.2 in-air undulator
Source size (FWHM, H × V)	412 µm × 6.2 µm	28 µm × 6.1 µm
Divergence (H × V)	11.5 µrad × 5.1 µrad	7.2 µrad × 5.1 µrad
Vertical focusing	11 white-beam Be CRL	11 white-beam Be CRL
Horizontal focusing	Multi-layer mirror (B_4_C/[Ru/B_4_C]_70_/Cr) (Δ*E*/*E* = 2.3%)	Be CRL
Monochromator	Si(111)	Si(111)
Photon energy	12.812 keV	12.812 keV
Beam size at the sample (FWHM, H × V)	18 µm × 14 µm†	15 µm diameter
Beam flux	2.0 × 10^13^ photons s^−1^ ‡	4.0 × 10^13^ photons s^−1^ §
Diffractometer	MD2S	MD2S¶
Sample changer	FlexHCD	FlexHCD
Detector	Dectris Eiger X 4M	Dectris Eiger X 4M

† Measured by scanning a tungsten wire through the beam.

‡ Measured with 100% transmission and 200 mA filling mode in the storage ring.

§ Estimated based on the new source characteristics.

¶ Refurbished.

**Table 2:** Diffraction data collection and refinement statistics for FAE and trypsin Numbers in parentheses are for the innermost and outermost shell, respectively.

Protein	FAE	Trypsin-EDNA	Trypsin-fast
Data collection			
Beamline	MASSIF-3	MASSIF-3	MASSIF-3
Energy (keV)	12.812	12.812	12.812
Oscillation range (°)	135	140	140
Increment (°)	0.05	0.1	0.2
Images per second	500	500	746
Exposure time per image (ms)	2.0	2.0	1.34
Total collection time (s)	5.4	2.8	0.93
Angular speed (° s^−1^)	25	50	149
Flux transmission (%)	1	1	3
Resolution range (Å)	48.9–1.80 (48.9–9.00, 1.84–1.80)	43.8–1.43 (43.8–5.54, 1.48–1.43)	43.8–1.43 (43.8–5.55, 1.48–1.43)
Space group	*P*2_1_2_1_2_1_	*P*2_1_2_1_2_1_	*P*2_1_2_1_2_1_
Unit-cell dimensions (Å)	65.5, 108.4, 112.9	54.3, 58.0, 66.9	54.3, 57.9, 66.9
Unique reflections	73998 (678, 4353)	39219 (781, 3669)	39193 (778, 3704)
*R*_meas_ (%)	11.7 (5.6, 97.9)	9.7 (4.3, 156.4)	10.0 (4.4, 171.9)
*R*_p.i.m._ (%)	5.0 (2.4, 41.6)	4.2 (1.9, 68.4)	4.3 (2.0, 75.1)
〈*I*/σ(*I*)〉	11.0 (32.2, 1.7)	11.4 (31.6, 0.9)	11.6 (32.3, 0.9)
σ_ano_	1.113 (2.402, 0.732)		
CC_1/2_ (%)	99.6 (99.8, 75.0)	99.8 (99.8, 47.0)	99.8 (99.8, 43.0)
Completeness (%)	98.5 (97.0, 98.4)	99.0 (99.5, 95.9)	99.1 (99.1, 97.2)
Multiplicity	5.2 (4.3, 5.3)	5.3 (4.8, 4.8)	5.3 (4.7, 4.9)
Wilson *B*-factor (Å^2^)	13.7	15.7	15.7

Phasing and refinement			
Resolution range (Å)	48.9–1.80	43.8–1.43	43.8–1.43
No. of reflections	70226	37260	37232
CC_weak_ / CC_all_	22.9 / 37.3		
Contrast original / inverted	0.242 / 0.864		
No. of automatically built residues			
*SHELXE*	529		
*ARP/WARP*	561		
*R*_work_ / *R*_free_ (%)	17.5 / 20.0	17.7 / 19.8	18.5 / 20.6
No. of protein atoms	4588	1629	1629
No. of water molecules	438	232	232
No. of ions and buffer molecules	8 Cd, 1 glycerol	1 Ca, 6 SO_4_, 1 benzamidine	1 Ca, 6 SO_4_, 1 benzamidine
Mean *B*	19.8	10.9	11.1
R.m.s. deviations			
Bond length (Å)	0.012	0.013	0.013
Bond angle (°)	1.77	1.78	1.78
PDB code	6y8g		6swv
